# Neural encoding of linguistic features during natural sentence reading

**DOI:** 10.1016/j.isci.2025.112798

**Published:** 2025-05-30

**Authors:** Vinay S. Raghavan, Lucas C. Parra

**Affiliations:** 1 Department of Biomedical Engineering, The City College of New York, New York, NY 10031, USA

**Keywords:** Neuroscience, Cognitive neuroscience, Linguistics

## Abstract

Reading is a complex process that involves translating characters into meaning. Orthographic, phonologic, and semantic features of individual words seem to play a role, but it is not clear how the brain encodes these features during natural reading. To answer this, we analyzed eye tracking and electroencephalography (EEG) signals while proficient adult readers read full sentences of English text. We found that fixation-locked neural responses are modulated by the complete linguistic hierarchy. This included modulation with phoneme and phonotactic information, typically observed during auditory processing of speech. Notably, responses were modulated by the phonotactics of prior words and phonemes of the current word. Our results indicate that natural sentence reading involves, in parallel, comprehensive encoding of the fixated word, phonotactic encoding for previous words, and contextual encoding of words around fixation.

## Highlights

• Written, spoken, and semantic features are encoded concurrently during reading
• Speech-related encoding lags behind the fixated words
• Stronger encoding of semantic context coincides with slower reading
• Results support the interactive account of reading

## Graphical abstract

**Figure:**
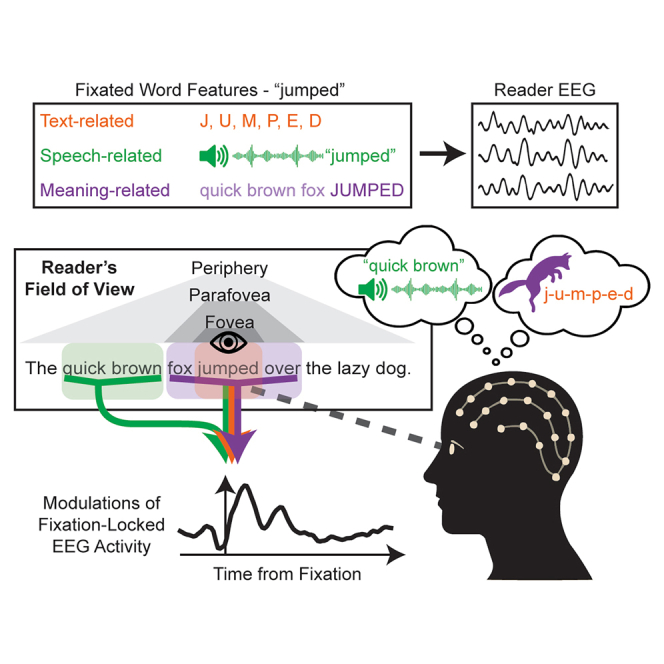

## Introduction

Humans can acquire the remarkable ability to read and comprehend written language. Studies of reading proficiency have pointed to the importance of the alphabetic principle, i.e., the association of written symbols with speech sounds, and phonologic coding.^1^^,^^2^^,^^3^ The evidence for the joint roles of phonologic and orthographic encoding comes from behavioral and eye movement studies during reading.^4^ Gaze contingent displays, in particular, have established a “perceptual span” of text around fixation that influences reading behavior.^5^ These and other studies have demonstrated clear evidence for parafoveal vision influencing reading.^6^ What is not clear is what type of neural processing occurs in the perceptual span and how much of that processing is related to previous and upcoming words.

Recent work has combined neural recordings with tracking of eye movements to shed light on the neural substrates of reading.^7^ These studies show that scalp potentials evoked by the visual presentation of letters or words are modulated by orthographic, phonologic, and semantic information.^8^^,^^9^ Some have gone further, aiming to create spatiotemporal maps of diverse linguistic encoding during reading either directly^10^^,^^11^^,^^12^^,^^13^ or via meta-analysis.^8^^,^^14^ These approaches support the notion that reading involves a recurrent interaction of neural representations of orthographic, phonologic, and semantic features, as opposed to a simple feedforward transformation from vision to meaning.^15^^,^^16^^,^^17^ While these studies have suggested an interaction between brain areas that process phonologic and semantic information with visual areas, we lack clarity on whether this neural activity occurs in parallel for multiple words during natural reading.

Indeed, most studies present words one at a time, a technique known as rapid serial visual presentation (RSVP), to prevent overlapping neural responses due to eye movements or changing visual stimuli.^18^^,^^19^ While RSVP paradigms have enabled many key discoveries about reading,^20^ this form of stimulus presentation strays far from natural reading.^21^ This has motivated more naturalistic reading paradigms, which offer many advantages for studying the brain.^22^^,^^23^ In particular, RSVP paradigms entirely ignore the debate over serial vs. parallel processing during reading.^24^ Readers skip about one-third of words during natural reading^4^; nevertheless, it remains unclear whether parafoveal information is processed automatically or involves covert shifts in attention. Rapid parallel visual presentation techniques, which show a small number of words at once, have been used to demonstrate parallel processing of three-word sentences.^25^^,^^26^ However, like RSVP paradigms, this approach lacks eye movements; therefore, it remains unclear when parallel linguistic encoding occurs relative to eye movements during natural reading.

To study the natural reading of full sentences, experimenters have captured eye gaze and neural activity using eye trackers and electroencephalography (EEG) to measure the average neural activity locked to fixations, known as the fixation-related potential (FRP).^23^^,^^27^^,^^28^ More recently, linear systems modeling was used to separate overlapping brain responses associated with multiple fixations during natural reading.^29^^,^^30^ We will refer to these as “fixation responses” and will favor this approach over conventional FRPs, which are confounded by the overlap and autocorrelation of eye movements. When compared with RSVP, active reading with eye movements enhances the benefit of visual previews and the influence of the word to the left of fixation.^31^ This benefit is not surprising given fundamental differences between visual processing with vs. without constrained eye movements.^32^ Nevertheless, studies with natural eye movements often use gaze-contingent displays rather than natural reading.^5^ To address this, recent studies used imperceptible flickering of individual words, which is detectable in magnetoencephalography. This technique showed evidence for parafoveal processing of lexical and semantic information.^33^^,^^34^ However, these studies used artificial sentence pairs to reveal a single contrast at a time, leaving it unclear how the complete linguistic hierarchy may be processed in parallel during natural reading.

To explore neural encoding of linguistic features during natural reading, we conduct a comprehensive analysis of neural activity while reading full sentences using co-registered eye-tracking and EEG data. We extracted orthographic, phonologic, and semantic features for each word and measured how fixation responses are modulated by these linguistic features. We used the same linear modeling formalism used for natural speech perception,^35^^,^^36^ referred to as temporal response functions (TRFs). If a feature significantly modulates EEG responses associated with the current fixation, we conclude that the brain must represent or “encode” this feature. To assess the parallel processing of multiple words, we measured modulations with linguistic features of words at and beyond the current fixation. Finally, we measured the correlation between the encoding of each linguistic feature and reading time. The results suggest that reading involves, at each fixation, a dynamic neural process encoding multiple features of the fixated word, phonotactic encoding for previous words, and contextual encoding in a window around fixation.

## Results

### Reading-related fixations modulate neural activity

**Figure 1:**
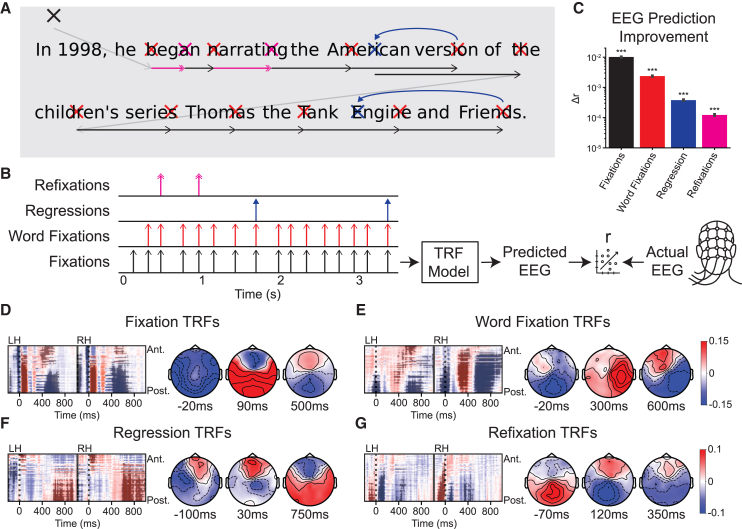
Analysis of reading-related fixations using temporal response functions (A) Example sentence and fixation locations. Sentences span 1–7 lines of text on the screen and are presented all at once. The black cross indicates a fixation anywhere but a word, red crosses indicate fixations on words, blue crosses indicate regressions (fixations on a previously fixated word), and pink crosses indicate refixations (fixations on a different position of the same word). Arrows indicate different types of saccades. (B) The time series of all fixations, word fixations, regressions, and refixations were used as predictive features to model EEG data. The model is a linear superposition of temporal response functions (TRFs) for each feature and each EEG electrode. (C) The change in Pearson correlation between the true and predicted EEG for each reading-related fixation (mean ± SE; ∗∗∗ for p_boot_ < 0.001). (D–G) TRFs represent modulations to fixation-locked neural responses with positive and negative values indicated in red and blue, respectively. Color bars for TRF magnitude, a.u., are shared between D and E and between F and G. Left and right correspond to electrodes covering the left and right hemispheres, and vertical axes are different electrodes sorted from anterior to posterior scalp locations (top to bottom). Each TRF captures additive contributions to the fixation response for specific types of fixations. Darker shading indicates significant clusters of electrodes and time points. Time points near the peaks of TRF clusters are plotted across the scalp.

We combined data from two datasets,^37^^,^^38^ resulting in a total of 29 participants who completed 41 sessions of reading 300–400 sentences (19.6 ± 9.4 words) from English Wikipedia articles and/or movie reviews (Figure 1A for an example sentence). First, we modeled EEG responses to blinks and saccades (Figure S1). We then modeled the additive contribution from fixations in general and different types of reading-related fixations, including fixations on words, regressions, and refixations (Figure 1B). To model EEG responses to these events, we used TRFs, i.e., the linear superposition of responses associated with each type of fixation. This method extracts temporally overlapping responses to possibly correlated discrete events or continuous signals from continuous EEG without the need for epoching or time-locked averaging.^39^^,^^40^ The TRF formalism is the same linear systems modeling used by others.^41^^,^^42^^,^^43^ The accuracy of the linear prediction is measured using conventional Pearson’s *r*, which captures here the temporal correlation between the predicted and the actual EEG time course and is computed separately for each electrode. To determine the contribution of each feature, we measured the increase in *r* from adding each fixation behavior as a predictor in order, i.e., fixations, word fixations, regressions, and refixations. The resulting changes in correlation were tested for significance with the hierarchical bootstrap, a multilevel non-parametric test.^44^ When averaged over all electrodes, we find significant increases in *r* for all four fixation-related behaviors averaged over all 105 electrodes (Wilcoxon signed-rank test, all *n* = 41; fixation: *W* = 861, *r* = 1.13, word fixation: *W* = 858, *r* = 1.10, regression: *W* = 840, *r* = 0.99, refixation: *W* = 806, *r* = 0.87; all *p*_boot_ < 0.001, *N*_boot_ = 10^4^) (Figure 1C).

To determine which time points and electrodes showed significant modulations to each behavior, we conducted a non-parametric spatiotemporal cluster permutation test on each TRF (*t*-statistic at threshold: *t* = 2.5) and identified clusters with permutation *p* < 0.05.^45^ We report broad spatiotemporal characteristics of each cluster and instead refer to the figures to ascertain the general extent of each cluster.^46^

We found that responses to fixations contain five clusters, including one before fixation onset, one around fixation onset, one just following fixation onset, and two much later (Figure 1D). Word fixation TRFs contained four clusters: one around fixation, as well as three later ones seen mainly in the right hemisphere (Figure 1E). Regression TRFs contained three clusters: one in the left hemisphere before fixation onset, one just after, and a broad one much later (Figure 1E). Refixation TRFs contained three clusters both before and after fixation (Figure 1F). Overall, these TRFs indicate pronounced neural responses to fixations during natural reading, as well as additive modulations to fixation responses that index a range of reading-related behaviors.

### Linguistic features of the fixated word modulate neural activity

**Figure 2:**
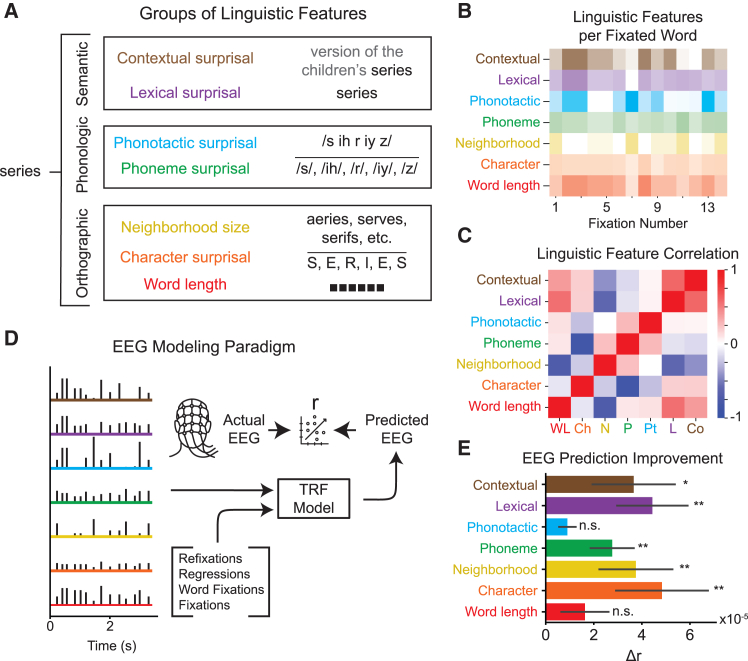
Analysis of linguistic feature encoding (A) Different categories of linguistic features for the example word “series.” There were seven such features for each word. (B) These linguistic features are extracted from each word that was fixated upon. (C) The correlation matrix of linguistic feature values of all fixated words (color bar indicates Pearson correlation). Certain linguistic features were found to be highly correlated, indicating the need to analyze each feature alongside all others. (D) Features were encoded as a time series locked to the word fixations, i.e., as pulse trains with height modulated by the feature value. These features were included as predictors alongside the previous fixation features to improve EEG response predictions. (E) The change in EEG prediction correlation from adding each feature (mean ± SE). Features that significantly improved EEG predictions are indicated with ∗ for *p*_boot_ < 0.05, ∗∗ for *p*_boot_ < 0.01, and n.s. for *p*_boot_ ≥ 0.05.

Next, we extracted orthographic, phonologic, and semantic “surprisal” features for each word (Figure 2A). Surprisal is the negative log likelihood of encountering a particular character/phoneme/word assuming statistical regularities of language.^47^ For example, “lexical surprisal” is defined as the negative log of the word probability in a large corpus of English text.^48^ We use surprisal because it is associated with a change in internal representations and with unexpected events, similar to prevalent EEG responses, e.g., mismatch negativity, error-related negativity, P300, and N400.^49^ Additionally, surprisal can also be seen as a measure of prediction error, which plays an important role in the “interactive model of reading.”^15^^,^^16^^,^^17^ In addition to lexical surprisal, we quantified character and phoneme surprisal, defined as the average surprisal of all the characters and phonemes in the word, respectively; phonotactic surprisal, defined as the surprisal of the word’s phoneme sequence given English phoneme constraints; and contextual surprisal, defined as the surprisal of the word given all previous words in the sentence using a GPT2 model. In addition to surprisal features, we extracted word length and neighborhood size, two features related to visual uncertainty that have been extensively studied in the context of reading.^50^ In total, we have 7 features organized into 3 groups: orthographic, including word length, character surprisal, and neighborhood size; phonologic, including phoneme surprisal and phonotactic surprisal; and semantic, including lexical surprisal and contextual surprisal.

We are interested in how these features change neural activity associated with the current fixation. Therefore, we evaluated these features for each fixated word (Figure 2B) and coded them as time-series vectors with delta peaks at fixation onset, scaled by the value of the corresponding feature (Figure 2D). To subtract the common response associated with other reading-related fixations, we also included as predictors the same fixation types as aforementioned (Figure 1B). Given this model setup, the corresponding TRFs encode an additive contribution to the fixation response that scales linearly with each of the seven linguistic features. To determine the unique contribution of each feature, we measured *r* using all linguistic features except the one of interest. We then added this feature to the model and measured how much *r* changed. This ensures that we measure changes in EEG prediction correlation due to the unique information contained in the feature of interest, rather than due to information that is shared between features (Figure 2C). The resulting changes in correlation were tested for significance with the hierarchical bootstrap.^44^

**Figure 3:**
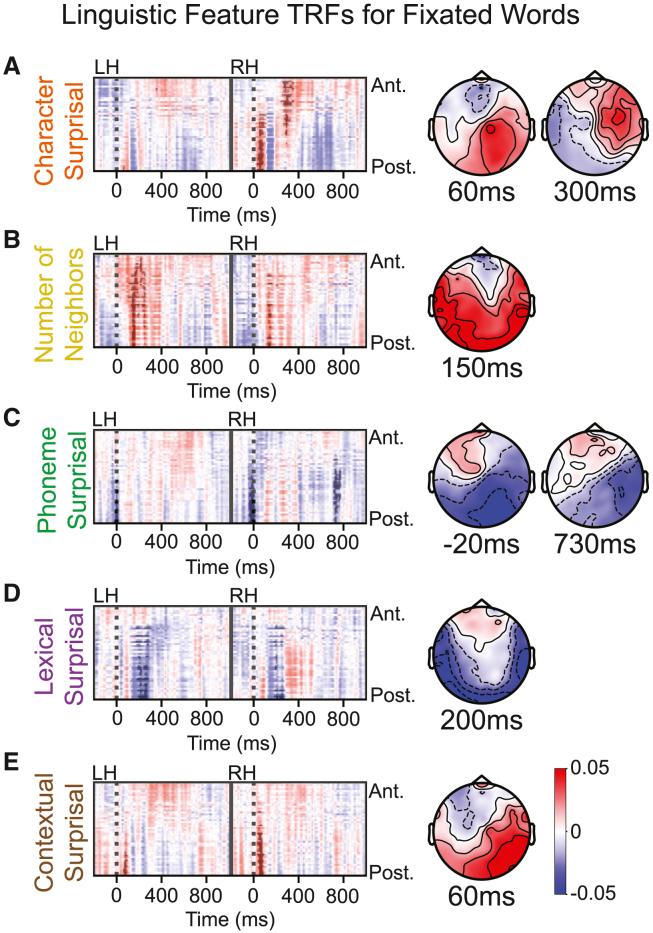
TRF results for each linguistic feature of the fixated word found to improve EEG predictions TRF images are split between left and right hemispheres and organized from anterior to posterior (top to bottom), with significant clusters shown darker than the surrounding TRF (*p* < 0.05; left) and TRF scalp maps for each feature near the peak of each significant TRF cluster (right) for character surprisal (A), neighborhood size (B), phoneme surprisal (C), lexical surprisal (D), and contextual surprisal (E). The same color bar for TRF magnitude (a.u.) is used for all plots.

A Wilcoxon signed-rank test was conducted to estimate the improvement in fixation response prediction. We found that character (*W* = 584, *r* = 0.35, *p*_boot_ = 0.0044), neighborhood (*W* = 651, *r* = 0.48, *p*_boot_ = 0.0042), phoneme (*W* = 631, *r* = 0.45, *p*_boot_ = 0.0032), and lexical (*W* = 670, *r* = 0.53, *p*_boot_ = 0.0004) information significantly modulated neural responses, while contextual (*W* = 597, *r* = 0.38, *p*_boot_ = 0.028) information weakly modulated neural responses. Word length (*W* = 363, *r* = 0.04, *p*_boot_ = 0.085) and phonotactic (*W* = 562, *r* = 0.31, *p*_boot_ = 0.0794) information were not found to modulate responses. Next, we analyzed the dynamics of neural responses associated with each of these features across time from fixation and over different electrodes using the cluster permutation test (Figure 3). For each feature, we show the associated TRF, with significant clusters shown darker than the surrounding TRF (*p* < 0.05). We also show scalp maps around the peak of each significant TRF cluster.

Responses to character surprisal show two right hemisphere clusters, one early and one late (Figure 3A). Responses to neighborhood size showed an early bilateral posterior cluster (Figure 3B). Responses to phoneme surprisal showed two clusters: one around fixation onset and the other much later in the right hemisphere (Figure 3C). Responses to lexical surprisal showed a bilateral posterior-lateral cluster (Figure 3D). Responses to contextual surprisal contain one early cluster, mainly in the right hemisphere (Figure 3E). Notably, we observe a cluster in the phoneme surprisal response occurring in right posterior electrodes before processing of the current fixation can occur. We also see a high degree of spatiotemporal overlap between clusters in the character and contextual surprisal responses, both of which follow closely after the first phoneme response cluster.

### Linguistic features of words left and right of fixation modulate neural activity

**Figure 4:**
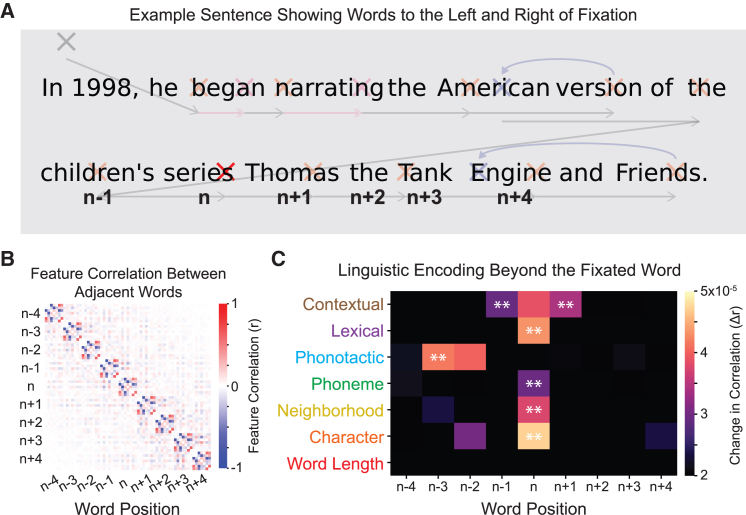
Analysis of linguistic feature encoding across word position (A) Word position approach for encoding linguistic information in an example sentence. Word “n” stands for the currently fixated word (red cross), whereas *n* − 1 and *n* + 1 are the left and right neighbors, etc. In this example, there is no *n* − 2, *n* − 3, or *n* − 4 because there is only a single word to the left on this line of text. (B) Correlation of features for currently fixated (diagonal blocks) and between neighboring words (off-diagonal blocks). The color bar indicates Pearson correlation. (C) Linguistic encoding across word position and fixation number, measured as the increase in Pearson correlation *r* between the real and predicted EEG responses when adding that feature to the model, as indicated by the color bar. Features are marked with ∗∗ for *p*_boot_ < 0.01 and with color for *p*_boot_ < 0.05 and Δ*r* > 2 × 10^−5^ (uncorrected).

Next, we aimed to extend the previous approach to study the parallel processing of linguistic information. We did this by modeling the linguistic features of each word to the left and right of fixation (Figure 4A). When comparing these predictors, we saw that linguistic features are correlated within words but uncorrelated between neighboring words (Figure 4B). We, therefore, model linguistic features for each word position individually. First, we model word positions in the region *n* − 4 … *n* … *n* + 4. The fixated word *n* is in the fovea, neighboring words *n* − 1 and *n* + 1 are in the parafovea, and the remaining words are in the periphery.^51^ This allowed us to measure the modulation of fixation-related neural activity with linguistic features of words across the reader’s field of view. Figure 4C shows the increase in Pearson correlation *r* for each feature in each word position and relative fixation, masked by features found to significantly increase the predicted EEG correlation (*p*_boot_ < 0.05 and Δr>2 × 10^−5^). The results for the fixated word (column *n* in Figure 4C) correspond to the previous results, i.e., Figures 3 and 4. Beyond those, we found that responses are modulated by phonotactic surprisal of words left of fixation (*n* − 3, *p* < 0.01). We also find that responses are modulated by contextual surprisal of the two neighboring words (*n* − 1, *n* + 1, *p* < 0.01), suggesting contextual processing in the parafovea.

### Phonotactic and contextual surprisal of surrounding words modulate neural activity

**Figure 5:**
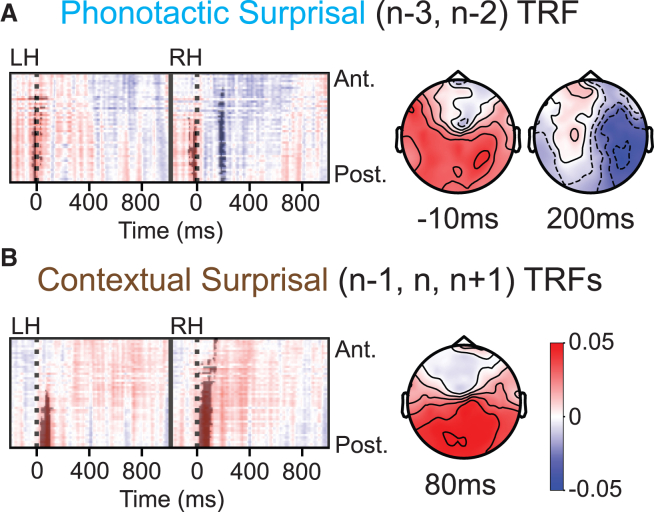
Linguistic feature responses for surrounding words (A) Phonotactic surprisal TRF averaged over word positions *n* − *3* and *n* − *2* (left) and EEG scalp maps at significant clusters (right). (B) Contextual surprisal TRFs (left) in a window around fixation (words *n* − *1*, *n*, and *n + 1*) and EEG scalp maps around the peak of each TRF cluster. Color bars for TRF magnitude, a.u., are shared between A and B.

Next, we analyze the TRFs for phonotactic and contextual surprisal of the relevant surrounding words. To simplify the analysis, we averaged the TRF across relevant word positions (with *p*_boot_ < 0.05 and Δ*r*>3 × 10^−5^). For phonotactic surprisal, this is *n* − *3* and *n* − *2* (Figure 5A), and for contextual surprisal, it is *n* − 1, *n*, and *n* + 1 (Figure 5B). When averaging over different positions, we assumed that the TRFs are similar across positions. We confirmed this visually on the TRFs computed separately for each position (Figure S2). We observed two clusters in the average phonotactic TRF: one bilateral around fixation and the other after fixation in the right hemisphere (Figure 5A). We observed one cluster in the average contextual TRF just after fixation in posterior electrodes (Figure 5B).

### Modulation with contextual surprisal correlates with reading speed

**Figure 6:**
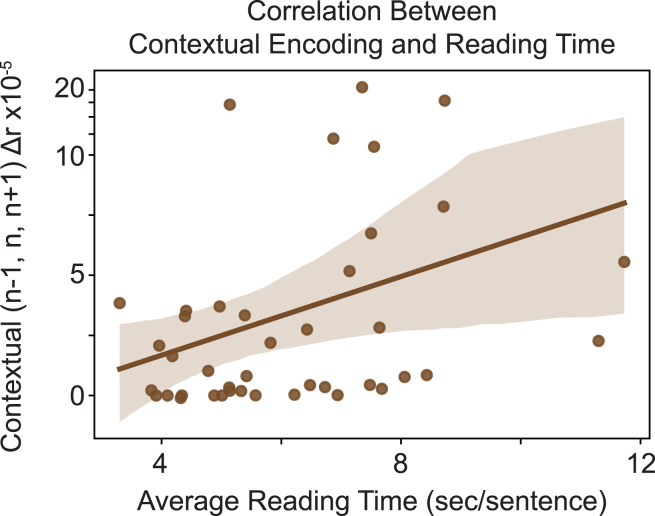
Average reading time vs. contextual encoding Change in EEG prediction correlation from contextual surprisal of words *n* − *1*, *n*, and *n* + *1* over reading sessions (linear fit ± 95% confidence interval).

Finally, we aimed to test for behavioral effects of neural encoding by measuring the correlation between reading time and the linguistic encoding of each feature, as measured by the increase in correlation between true and predicted EEG. We found that the average change in EEG prediction correlation with contextual surprisal over words *n* − *1*, *n*, and *n* + *1* was significantly correlated with reading time across reading sessions (Spearman *r* = 0.42, *n* = 41, *p* = 0.036, Bonferroni corrected; Figure 6).

## Discussion

In this study, we analyzed neural encoding of linguistic features of words during natural sentence reading. This analysis first revealed large modulations to reading-related fixation responses in the EEG, with considerable responses before and after fixation onset. Next, we observed modulations of fixation responses based on how surprising words were in terms of their character, neighborhood, phoneme, lexical, and contextual features. Finally, we found a modulation of fixation responses to phonotactic surprise of words in the left periphery and to contextual surprise of neighboring words (immediately left and right of fixation). Together, these results suggest dynamic neural processing of orthographic, phonologic, and semantic features upon fixation of each word.

Traditional EEG studies of reading focused on individual event-related potential components, such as the N400. The N400 evoked response is observed when the stimulus is surprising, e.g., the word is unexpected given the preceding sentence, is in an unrelated category, or has a large neighborhood size.^20^ If these evoked responses are modulated, it means that the brain encodes these linguistic features. However, many features are observed to modulate the N400, some of which are highly correlated. In the present analysis, we have extracted an entire spectrum of linguistic features and measured unique modulation of responses with each corresponding surprisal metric. Furthermore, the analysis method allowed us to study continuous natural reading, thus breaking away from the artificial constraints of discrete stimuli required for event-related analysis.

### Reading-specific neural activity locked to fixation

The current analysis method has previously been used to shed light on linguistic encoding during speech perception.^35^^,^^36^^,^^52^^,^^53^^,^^54^ Reading differs from speech perception in that readers must overtly select the stimulus. Here, the analysis revealed that fixation responses are strongly modulated when a fixation falls upon a word and further modulated by specific reading-related fixations. This fixation response holds the potential to shed light on the brain processes involved in reading, much as the envelope-tracking response has done for the neuroscience of speech.

Responses to fixations on words were significantly lateralized to the right hemisphere, suggesting that these responses may relate more to visuospatial attention.^55^ In contrast, refixation and regression responses were generally bilateral, with early regression responses appearing in frontal areas, suggesting some language- or saccade-related planning. Responses occurring before fixation may also be caused by the reliable differences in saccade amplitude and direction occurring with refixations and regressions, respectively. Additionally, refixation responses contained an occipital response around 100 ms with an opposite sign to that observed in the fixation response. One reason for this could be that the incoming saccades of refixations are generally lower in amplitude relative to other fixations, and saccade amplitude is known to modulate fixation responses around 100 ms.^56^

### Linguistic encoding upon fixation

**Figure 7:**
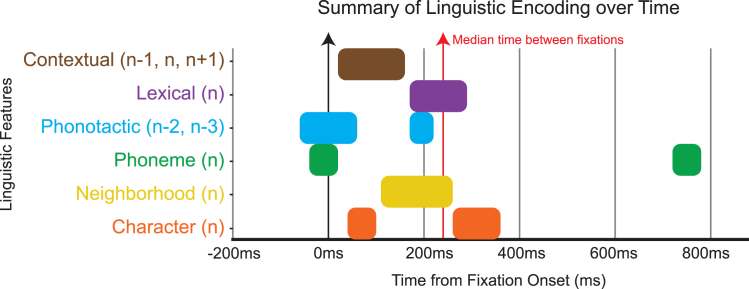
Summary of linguistic encoding Approximate encoding of linguistic features (*y* axis) over time from fixation onset (*x* axis). Blocks for each feature are shown over the time range of significant clusters when >1 channel was included in the cluster. The contextual feature cluster is taken from the average TRF of words *n* − *1*, *n*, and *n + 1*. The phonotactic feature clusters are taken from the average TRF of words *n* − *2* and *n* − *3*. All other clusters are taken from the TRFs of word *n*.

Upon fixation, we also see a cascade of neural activity associated with the linguistic features of each word, summarized in Figure 7. This begins with responses to phonotactic surprisal of words left of fixation occurring in the period around fixation (−10 ms, cyan). We also observe a response to phoneme surprisal of the fixated word around fixation (−20 ms, green). This response is observed before bottom-up processing of the fixated word can occur, suggesting that predictive processing of the fixated word may occur through speech-related pathways.

Next, we see the modulation of occipital areas due to character and contextual surprisal of the fixated word, as well as contextual surprisal of words in the parafovea (∼60 ms, orange and brown). This indicates that character and contextual information are rapidly recognized upon fixation, and this low-level visual contextual processing likely supports the skipping of words during natural reading.^57^^,^^58^

Following these initial responses, we see modulations due to neighborhood size (∼150 ms, yellow) and lexical surprisal (∼200 ms, purple). Both responses are similar in timing to a response to phonotactic surprisal (∼200 ms, cyan), indicating a potential role of speech-related processing of previous words in priming the lexical identification of the current word.^1^

We see further modulations due to character surprisal (∼300 ms, orange), possibly revisited after the lexical identification. Much later, we see responses to phoneme surprisal (∼730 ms, green), which is consistent with the delayed phonotactic processing of words to the left. These last two responses occur after the reader has already advanced to the next fixation.

Altogether, this pattern of response indicates a highly interactive process of reading. At first, low-level speech-related representations are generated by top-down predictions. Next, low-level visual and contextual features are processed concurrently. Finally, high-level lexical identification is checked against low-level visual input. Regardless of this interpretation, the overall temporal pattern of Figure 7 is consistent with a recurrent neural dynamic that combines bottom-up and top-down processing, as proposed in the “interactive account” of reading.^15^^,^^17^

### Phonotactic encoding occurs to the left of fixation

Activity locked to fixations is traditionally thought to relate to visual processing.^28^^,^^59^ Here, we found, however, that fixation responses were modulated by phonologic features, which are more commonly linked to auditory-motor processing of speech.^52^^,^^53^^,^^60^ This is particularly remarkable given that these fixation responses (for word *n*) were modulated by phonotactic features of words that are no longer in foveal or parafoveal vision (words *n* − 2, *n* − 3). This observed spatial shift between fixation and phonotactic encoding suggests a delay between eye movements and higher-level speech-related processing of the text, as though there is a lag between reading a word and knowing if it “sounded right.” This is in line with the eye-voice span during reading aloud or the distance between the fixated and pronounced word at any given point in time, which was found to be approximately 15 letters, 2 to 3 words, or 500 ms.^61^

Not only does this indicate a delay in speech-related processing during reading but it also indicates that eye movements play a role in modulating speech-related processing. Indeed, previous work has indicated transient amplification of auditory processing in the period immediately preceding fixation,^62^ and there is convergent evidence for a common linguistic processing rate between speech syllables and reading fixations.^63^

### Contextual encoding occurs in a window in parallel

We observe modulations in response to contextual surprisal for the word at fixation as well as the words immediately to the left and right of fixation. This suggests that contextual information in sentences can be encoded through parafoveal processing, in agreement with past studies.^26^^,^^34^ Additionally, the early and overlapping responses observed to contextual surprisal of all words in this window indicate that contextual information is rapidly processed in parallel upon fixation. This suggests that parafoveal processing is likely to occur through automatic processing, potentially without attention, rather than through covert shifts in attention. Together, this suggests that processing in the perceptual span is based on contextual information rather than low-level visual information.

A previous study of the difference between reading word lists with and without eye movements indicated that eye movements increase parafoveal previewing to the right of fixation and lexical processing to the left of fixation.^31^ This indicates that modulations from contextual information of words *n* − *1* and *n* + *1* might only be observed during natural reading with eye movements. Additionally, these effects are observed in the 160–300 ms time window after fixation onset, suggesting that early parafoveal preview may affect later responses, such as more typical N400 responses. Similarly, preview manipulation during sentence reading with eye movements showed responses to invalid previews around 230–300 ms.^29^ In this study of natural sentence reading with eye movements, we see responses to lexical and contextual information typically associated with the N400 occurring much earlier, including responses to contextual information in the parafovea. This suggests that typical N400 responses may not occur during natural reading since parafoveal information may facilitate that type of processing, thereby altering its temporal dynamics. Alternately, because the N400 is sensitive to many linguistic measures, it is also possible that the N400 response is captured by the shared variance between features, which we chose not to analyze since it cannot be attributed to any single feature.

### Contextual encoding correlates with reading time

Finally, we observed that reading time correlates with the neural encoding of contextual surprisal. Specifically, readers who spend more time reading show larger neural responses to contextual surprisal. This could suggest that reading slowly is associated with more contextual integration. Another explanation could be that slower readers require a greater amount of contextual processing to accomplish the same reading process. A further explanation could be that slower readers show a more consistent timing between fixation and contextual processing, leading to better linear TRF predictions.

Recent evidence found that parafoveal previewing of the upcoming word was decreased for more contextually surprising words, and this effect was greater for slower readers.^34^ This agrees with our result that slower readers show more contextual processing but also indicates less previewing of high surprisal words in the parafovea.

### Future work

The encoding model paradigm has been used very successfully to study the neuroscience of speech. Bringing it to the study of natural reading holds enormous potential. Specifically, natural reading experiments can enable more ecologically valid findings through more straightforward data collection procedures, enabling a whole range of new types of analyses. This includes analyses of character-level information or the relative position of the fixation with each word.

This same analysis paradigm could be used with separate populations to uncover differences in linguistic encoding. For example, this could include children at different ages to study reading acquisition, as was done to test phonetic encoding during speech perception.^64^^,^^65^ A similar comparison could be made in language learners to assess proficiency^66^ or in aging populations to assess changes in cognition.^67^ This approach could also serve as a key tool to understand how the neural activity during reading differs in individuals with dyslexia, developmental language disorders, or other disorders that indirectly affect reading, such as schizophrenia or autism spectrum disorder.^68^^,^^69^^,^^70^^,^^71^

Alternatively, one could use the approach to study the effects of different reading tasks. For example, the data we analyzed here involved natural reading to answer comprehension questions. Thus, participants took more time as compared to speeded reading with the goal of identifying specific relation types in the text.^37^^,^^38^ Making explicit judgments from a text is a different task goal and may result in different reading behaviors, such as more parafoveal processing to enable more skips. Future work could investigate different tasks during reading in a range of contexts.

The TRF methodology is not specific to neural data and can be used to model a range of linear stimulus-response mappings. Therefore, this same technique could be applied directly to eye-tracking data, such that it could be possible to determine the influence of linguistic information of words in the parafovea and periphery on eye movements while still controlling for overlapping responses and the shared variance between correlated features.

### Limitations of the study

This study addressed the neural encoding of linguistic information measured at the word level for words left and right of the fixated word. However, this analysis does not account for the position of fixation within the word or the length of adjacent words, which are known to influence eye movement properties,^72^ the processing of words in the parafovea,^73^ and parallel lexical processing.^74^ Future studies could investigate parafoveal processing by modeling information at more fine-grained levels, such as the bigram, character, or visual level,^75^ or by separately modeling fixations depending on where within each word the fixation occurs. Nevertheless, these results provide a representation of how parallel encoding of words occurs *on average* during natural sentence reading.

The TRF methodology assumes a consistent timing and linear scaling between feature input and neural responses; however, some models of reading allow for differing timing of lexical access depending on characteristics, such as word frequency.^16^ Therefore, models that can account for some of these effects, such as dynamic warping or spline regression, may prove insightful.^43^^,^^76^

Additionally, the spatial topographies of TRF time points are highly influenced by the method of EEG referencing used. In this case, all data were referenced to Cz, making it challenging to meaningfully interpret the TRF topographies. Nevertheless, the timings of significant TRF components remain interpretable to measure the unfolding of linguistic processing across time from fixation onset (Figure 7).

The analysis of linguistic encoding across word positions was designed to test for parafoveal or peripheral processing of linguistic information. As such, this analysis explicitly removed words that spanned different lines. However, our result indicating that phonotactic encoding lags behind suggests a buffer that is likely to span lines. Future work could compare the influence of linguistic features of words across lines of text.

Furthermore, because the linguistic features analyzed were so highly correlated, we aimed to identify effects driven uniquely by each feature of interest. While it is possible that there are effects driven by the shared variance between these features, this analysis is not designed to reveal those effects. This analysis, therefore, may even have a conservative bias due to the potential masking effect caused by the removal of shared variance.

It is also important to note that the measure of contextual surprisal used here represents many types of information simultaneously. Because this measure simply captures how unexpected a word is given all previous words in the sentence, it can capture grammatical and syntactic constraints, as well as higher-level semantic relevance. Therefore, it is unclear which or how many aspects of contextual surprisal are represented by the observed modulations. Future studies could tease apart each of these features.

Finally, all text materials and analysis methods were based on the English language, and all participants had English as their native language; therefore, future studies should aim to understand how this approach translates to other languages.^77^

## Resource availability

### Lead contact

Further information and requests for resources and materials should be directed to and will be fulfilled by the lead contact, Vinay S. Raghavan (vraghavan@ccny.cuny.edu).

### Materials availability

This study did not generate new materials.

### Data and code availability

All original datasets are publicly available (https://osf.io/q3zws/ and https://osf.io/2urht/). This paper does not report original code. Any additional information required to reanalyze the data reported in this paper is available from the lead contact upon request.

## Acknowledgments

The authors acknowledge support from the 10.13039/100000183Army Research Office (ARO WF911-NF-24-1-0031) for funding their primary research activities. However, the research presented in this paper was conducted independently and was not directly supported by ARO funding.

## Author contributions

Conceptualization, V.S.R.; methodology, V.S.R. and L.C.P.; investigation, V.S.R.; visualization, V.S.R.; supervision, L.C.P.; writing – original draft, V.S.R. and L.C.P.; writing – review and editing, V.S.R. and L.C.P.

## Declaration of interests

The authors declare no competing interests.

## STAR★Methods

### Key resources table

**Table:** 

REAGENT or RESOURCE	SOURCE	IDENTIFIER
**Deposited data**		

ZuCo Dataset	OSF	https://osf.io/q3zws/
ZuCo 2.0 Dataset	OSF	https://osf.io/2urht/

**Software and algorithms**		

naplib-python 2.5	Neural Acoustic Processing Lab, New York City, USA	https://github.com/naplab/naplib-python
wordfreq 3.1.1	Robyn Speer	https://github.com/rspeer/wordfreq
python-BLICK 0.2.12	Michael McAuliffe	https://github.com/mmcauliffe/python-BLICK/
transformers 4.39.3	Hugging Face, New York City, USA	https://github.com/huggingface/transformers; RRID:SCR_020958
MNE-Python 1.9.0	MNE-tools	https://github.com/mne-tools/mne-python; RRID:SCR_005972
CMU Pronouncing Dictionary 0.7b	Carnegie Mellon University, Pittsburgh, USA	http://www.speech.cs.cmu.edu/cgi-bin/cmudict

### Experimental model and study participant details

This study utilizes the ZuCo 1.0 and ZuCo 2.0 datasets.^37^^,^^38^ These two openly available datasets contain co-registered EEG and eye-tracking data from participants reading English sentences. Some sentences were part of the task-specific reading condition, involving the recognition of a specific relation in the sentence; others were part of normal reading conditions in which participants were asked to read the sentences and answer a control question afterward. Only data from the normal reading conditions were included in this study. These data were originally collected to facilitate research in natural language processing through the potential to use cognitive features to train and evaluate models.

The ZuCo 1.0 dataset contains recordings from 12 native English-speaking participants (5 female, 22–54 years, all right-handed). The ZuCo 2.0 dataset contains recordings from 18 native English-speaking participants (10 female, 23–52 years, 16 right-handed). All participants in both datasets participated in a linguistic assessment using the LexTALE test to gauge their vocabulary and language proficiency. All participants gave their written informed consent prior to participation in each of these studies, and both studies were approved by the Ethics Commission of the University of Zurich. Data from all participants in ZuCo 2.0 were included in the study, while participant YAC from ZuCo 1.0 was excluded due to poor data quality. Thus, we analyzed data from 29 participants total as a single experimental group.

### Method details

#### Experiment materials

The ZuCo 1.0 normal-reading conditions include 300 sentences randomly selected from the Wikipedia relation extraction dataset and 400 sentences from the Stanford Sentiment Treebank with roughly balanced sentiments (123 neutral, 137 negative, 140 positive). The ZuCo 2.0 normal-reading condition includes 349 sentences selected from the Wikipedia relation extraction dataset, 100 of which were also included in the ZuCo 1.0 datasets. This data was originally collected to aid automatic labeling of text for machine learning.

In both datasets, each sentence was individually presented to participants on a computer screen. The text was black, size 20-point Arial font on a light gray background, resulting in a letter height of 0.8 cm or 0.674° of visual angle. Sentences had anywhere from 3 to 65 words (mean: 19.6, s.d.: 9.4). a maximum of 80 letters or 13 words per line, and a maximum of 7 lines.

#### Experiment procedure

In ZuCo 1.0, each participant read the entire set of materials in two sessions of 2–3 h each on the same day. In the first session, they completed the Wikipedia sentences and half of the Stanford Sentences, and in the second, they completed the task-specific reading and the second half of the Stanford sentences. Each condition was preceded by a practice round of 3–5 sentences.

In ZuCo 2.0, each participant read the materials in one session of 100–180 min. The normal-reading and task-specific reading conditions were presented as 14 blocks, with the conditions alternating with each block. Each block was preceded by a practice round of 3 sentences.

In both datasets, the sentences were presented in identical order for each participant. Participants read the sentences at their own pace and used a control pad to advance to the next sentence and answer the control questions, allowing for natural reading behavior.

#### Data acquisition

In both datasets, data acquisition occurred in a sound-attenuated dark experiment room. Participants were seated with a chin rest 68 cm in front of a 24-inch monitor with a screen resolution of 800 × 600.

Eye position was recorded with an infrared video-based eye tracker (EyeLink 1000 Plus, SR Research) at a sampling rate of 500 Hz and an instrumental spatial resolution <0.01°. The eye tracker was calibrated with a 9-point grid before and repeatedly throughout the experiments.

EEG data were recorded using a 128-channel EEG Geodesic Hydrocel system (Electrical Geodesics) at a sampling rate of 500 Hz with a bandpass filter of 0.1–100 Hz. The recording reference was at Cz. The electrode impedance levels were checked before recording and kept below 40 kOhm.

#### Data pre-processing

The eye-tracking data consists of the horizontal and vertical position of the participants' gaze through time. Coordinates were given in pixels with respect to the monitor coordinates. While these raw data are provided, the EyeLink 1000 tracker further processed this eye position data to identify saccades, fixations, and blinks. Saccades were detected using the default parameters of the EyeLink: an acceleration threshold of 8000° per sec^2^, a velocity threshold of 30° per sec, and a deflection threshold of 0.1°. Fixations were defined as time periods without saccades with non-zero pupil diameter longer than 100 ms, and blinks were defined as periods without saccades in which the pupil diameter was zero or invalid. Therefore, we obtained the onset time of each blink, saccade, and fixation, which were encoded as a binary time series sampled at 100 Hz with a 1 at each event onset and 0 otherwise. Additionally, the gaze positions of each fixation were paired with the word bounding boxes to determine which (if any) word was in the participant’s gaze upon each fixation.

The EEG data were preprocessed using Automagic (version 1.4.6), a tool for automatic EEG data cleaning and validation.^78^ Of the 128 electrodes originally recorded, 105 scalp EEG channels were retained, 9 EOG channels were used for artifact removal using instantaneous linear regression, and 14 channels lying on the neck and face were discarded. Bad channels were identified and interpolated, and the Multiple Artifact Rejection Algorithm (MARA) was used to remove artifactual ICA components. Our only preprocessing steps beyond those done by Hollenstein and colleagues were to low-pass filter the EEG data with a 5th-order Butterworth filter with a cutoff at 30 Hz and downsample the EEG to a sampling rate of 100 Hz.

#### Reading-specific fixation features

With the complete information about all fixations in the dataset, we defined three additional fixation-related features: word fixations, refixations, and regressions. Word fixations are any fixation with a gaze location within a word’s bounding box. Word fixations account for 92.0% of the total fixations. Refixations are word fixations in which the currently fixated word is immediately fixated again. Refixations account for 14.3% of the total fixations. Regressions are any word fixation in which the currently fixated word has been fixated before but wasn’t the previously fixated word. Regressions account for 21.4% of the total fixations. Each type of fixation was encoded as a binary time series with a 1 at the fixation onset and 0 otherwise.

#### Word features

We extracted seven features of each word in the dataset: word length, average character unigram surprisal, orthographic neighborhood size, average phoneme unigram surprisal, phonotactic surprisal, word surprisal, and contextual surprisal. Each feature was encoded as a time series of impulses with the amplitude of the impulse given by the value of the feature and the timing of the impulse given by the fixation onset of the fixated word.

In addition to encoding the value of the fixated word, *n*, we also encoded the values of the features of the surrounding 8 words *n* − *4*, *n* − *3*, …, *n* + *3*, *n* + *4*, such that the amplitude of the impulse is given by the surrounding word, *n* + *a*, but the timing of the impulse is still given by the fixated word, *n*. These values were only encoded if the surrounding words were on the same line of text as the fixated words.^79^

To extract these features, we used the English portion of the *wordfreq* library for Python, containing a list of all the words in the Exquisite Corpus and their probabilities.^48^

#### Orthographic features

The word length feature is defined as the base-10 logarithm of the number of characters in the word. The number of characters per word ranged from 1 to 29 (mean: 6.24, s.d.: 2.96), leading to values of the word length feature ranging from 0 to 1.46 (mean: 0.74, s.d.: 0.22).

The average character unigram surprisal is defined as the mean surprisal of each character in the word. To compute the surprisal of each character, we first estimate the probability of each character by counting the occurrences of each character in each word in the word list, normalized by the word probability. The surprisal of each character is then computed as the negative base-10 logarithm of the probability of each letter. This provides high values for uncommon letters, such as “X” and “Q,” and low values for common letters, such as “E” and “S.” For each word, these values ranged from 0.93 to 4.18 (mean: 1.30, s.d.: 0.47).

The orthographic neighborhood size, or simply neighborhood size, is defined as the base-10 logarithm of the number of words that can be formed by changing exactly one letter in a word. To compute the neighborhood size for each word, we directly swapped each letter with every other letter of the alphabet and counted the number of words formed that were also present in the corpus. This provides high values for words with many orthographically close words, e.g., “love”: “cove,” “wove,” “live,” “lore,” etc., and low values for words with more unique letter combinations, e.g., “unwieldy.” These values ranged from 0 to 1.88 (mean: 0.81, s.d.: 0.72).

#### Phonologic features

To extract phonemes from words, we used the open-source CMU Pronouncing Dictionary and removed all stress information. Any words not found in the dictionary were estimated using the CMU LOGIOS Lexicon Generation Tool and manually checked.

The average phoneme unigram surprisal is defined as the mean surprisal of each phoneme in the word. To compute surprisal, we first estimate the probability of each phoneme by counting the occurrences of each phoneme in each word of the word list, normalized by the word probability. Surprisal is then computed as the negative base-10 logarithm of the probability of each phoneme. This provides high values for uncommon phonemes, such as/OY/and/ZH/, and low values for common phonemes, such as/AH/and/T/. These values ranged from 1.03 to 2.28 (mean: 1.39, s.d.: 0.15).

The phonotactic surprisal is defined as the negative logarithm of the likelihood that the word, as given by the sequence of phonemes, is well-formed in its language, as given by the BLICK algorithm.^80^ BLICK works by constructing maxent grammars consisting of weighted phonologic constraints. This provides a high value for poorly formed words, such as “Choquart” and “Knoxville,” and a low value for well-formed words, such as “speck” or “class.” These values ranged from 0 to 41.84 (mean: 3.84, s.d.: 4.04).

#### Semantic features

The lexical surprisal is defined as the negative base-10 logarithm of the word probability. This provides a high value for uncommon words, such as “malaise” or “torpedo,” and a low value for common words, such as “great” or “walk.” These values ranged from 1.27 to 9.0 (mean: 4.15, s.d.: 1.49).

The contextual surprisal is defined as the negative base-10 logarithm of the GPT-2 probability of the current word, given all previous words in the sentence as context. To compute this, we accessed the pre-trained OpenAI GPT-2 model and tokenizer hosted by HuggingFace using the *transformer* library for Python.^81^ For each sentence, we first used a period and space as input and found the probability of seeing the first word as the first word of a sentence. For each subsequent word, we used the period, space, and all previous words in the sentence as input and found the probability of seeing the current word given the sentence context. This provides a high value for contextually unexpected words, such as “she smelled the *envelope,*” and a low value for contextually expected words, such as “she smelled the *rose*.” These values ranged from 3.3∗10^−5^ to 14.02 (mean: 2.91, s.d.: 1.95).

#### Encoding models for neural representation

To determine how different ocular behaviors and linguistic features are represented in a reader’s brain, we used linear encoding models to predict participants’ neural activity as output using behavioral events and linguistic features as input.^40^ Specifically, we utilize banded ridge models to train regularized linear models that predict EEG responses using a time-lagged representation of the input.^82^^,^^83^ Note that the linear systems identification (modeling) formalism we used here can separate responses to generic fixations from the differential effect of more specific features, provided they are not collinear. For instance, the time series encoding for word fixations (Figure 1B, red) is not collinear with the time series encoding for all fixations (Figure 1B, black) because there are a few fixations that do not point to a word. This allows one to identify the added contributions of words to the fixation responses that are extracted at TRFs.

#### Banded ridge modeling

Ridge regression is typically used in encoding models to predict neural responses from stimulus features because regularization is needed to prevent overfitting due to limited amounts of data, noise in the neural data.^39^ However, it is not immediately clear how encoding models that combine feature spaces should approach the regularization of each feature in the input. Most prior work with encoding models that combine feature spaces uses a single regularization parameter for the entire combined feature space.^35^^,^^36^ Yet, feature spaces with different units, different numbers of dimensions, different degrees of correlation, different degrees of sparsity, and/or different degrees of predictive power will require different regularization parameters to prevent overfitting.^84^

To handle the problem of combining feature spaces, we turn to banded ridge regression. Note that we generally refer to this as “banded ridge” or “banded ridge modeling” to prevent confusion with the reading term “regression,” meaning a fixation on a previously fixated word. Banded ridge formalizes a method for setting separate regularization values, λ, for each feature space, leading to better prediction accuracy and interpretability.^83^ To determine the λ for each feature space, a cross-validation procedure is used, involving a search over each set of λ values for each feature space. This search is effective for finding the λ values for each feature space that allow each variable to fully explain its unique variance while distributing the shared variance between feature spaces. However, the distribution of the shared variance is ultimately dependent on minor details, such as the spacing of the λ search, and the search requires N_λ_^N_feature^ cross-validations, which can be computationally intractable for certain datasets and/or analyses.

To alleviate these problems, we utilize a progressive form of banded ridge that adjusts λ for each feature (on left-out cross-validation data) *in a pre-specified order.* With this approach, any shared variance will be attributed to the feature added first. This is implemented by sweeping the λ_1_ for the first feature, identifying the λ_1_ that maximizes the average cross-validated correlation between the true and predicted neural responses, and freezing λ_1_ for the first feature. Then, the next feature is concatenated to the input, and again λ_2_ is swept for the second feature while holding λ_1_ constant to identify the λ_2_ that maximizes that prediction correlation. This process is repeated for each additional feature.

This ordered search requires N_λ_∗N_feature_ cross-validations, alleviating some of the computational complexity of adjusting for λ_i_ all features independently. Nevertheless, this approach is potentially biased, as it will attribute all shared variance to the earlier feature and, thus, is dependent on the order of the features the experimenter chooses. Therefore, it is an effective method for reducing the computation required to determine the different optimal λ values for input features that are generally uncorrelated or when the shared variance between low-level and high-level features is more clearly attributable to low-level features.

To interpret only the unique variance of a specific feature in an unbiased manner, we can utilize progressive banded ridge with all features, but only assess the predictive power of the last feature added to the model. This removes the bias of feature order and requires N_λ_∗N_feature_^2^ cross validations–a useful reduction relative to the exhaustive search.

#### Banded ridge model structure

All models were trained using the sklearn Ridge model solved via the Cholesky decomposition and cross-validated with leave-one-block-out cross-validation to prevent artifacts caused by concatenating data across blocks.^85^ Each block contained about 50 sentences, and blocks were defined using the segments of data provided: ZuCo 1.0 had 6 segments of Wikipedia reading and 8 segments of sentiment reading, and ZuCo 2.0 had 7 segments of Wikipedia reading. Specifically, a model was trained using the data from each block, and during testing, the models from all blocks except the test block were averaged and used to predict the test response. All TRF models were trained with −0.2 to 1.0 s of delays, and 15 λ values were tested on a log scale from 10^0^ to 10^4^ for each feature for each participant. The same λ was used for all channels for a given feature. Regularization was applied by dividing the input features by their corresponding λ, as this is equivalent to multiplying the regularization term with λ.^82^

First, we assessed the predictive power of the ocular and fixation-related features using the progressive banded ridge model. The feature order was as follows: blinks, saccades, fixations, word fixations, regressions, refixations. This allowed us to determine the specific responses associated with fixations on words separate from fixations in general and those of regressions and refixations separate from word fixations in general. These features together form the baseline model.

Next, we assessed the predictive power of each word feature for the fixated word, *n*, and the 8 surrounding words *n* − *4*, *n* − *3*, …, *n* + *3*, *n* + *4*. This was done by concatenating all seven word-features for each word position to the baseline model in different orders such that each feature was used as the final feature. This allowed us to assess the unique variance explained by each word feature, independent from any shared variance with other features.

#### Temporal response function analysis

The weights of the linear encoding model that map time-lagged stimulus features onto brain responses are known as temporal response functions (TRFs). We plotted the TRFs of the behavioral features by reshaping the weights into an n_channels × n_time matrix. We then split channels in the left and right hemispheres and ordered the rows of this matrix from posterior to anterior (Figures 1D–1G, 3, and 5). This provides a sense of what neural activity occurs at what time and in what part of the brain in response to each of these features. MNE-Python was used to plot TRF time-steps on the scalp.^86^ Topographic maps were generated using electrode information provided in the original datasets, i.e., the Hydrocel-128 montage with corresponding neck and face channels removed.

### Quantification and statistical analysis

#### Change in EEG prediction correlation

To assess the predictive power of the first feature, i.e., blinks, we computed the average Pearson correlation between the predicted neural response and the true neural response at each channel for each cross-validated block for each participant. To assess each of the following features, we measured the change in Pearson correlation between the predicted and true neural responses when the additional feature was added. This provides a metric of how much additional variance each feature explains. To estimate the size of this effect, we report the one-sided Wilcoxon signed-rank test between the EEG prediction correlation averaged over electrodes before vs. after adding the feature for all reading sessions (*N* = 41).

#### Hierarchical bootstrap statistics

To determine whether the predictive power of each feature was significant, we employed the hierarchical bootstrap to account for the non-independence of results between sessions and blocks.^44^ This method leverages the fact that our data is hierarchically organized by participants, sessions, and blocks to compute bootstrapped statistics at each level of the hierarchy to keep the Type-I error rate low while retaining more statistical power than averaging over blocks. To test for overall feature encoding, we performed a hierarchical bootstrap of the prediction correlation averaged over all electrodes at the levels of participants, sessions, and blocks. All tests used *N*_boot_ = 10^4^ bootstraps.

#### Spatiotemporal cluster permutation test

We used MNE-Python to perform spatiotemporal cluster permutation tests.^45^^,^^86^ We conducted spatiotemporal cluster permutation tests on the TRFs across sessions using the adjacency map of the Hydrocel-128 EEG montage to compute spatial proximity in addition to the temporal proximity of cluster components. We identified clusters using a threshold of |*t*|> = 2.5 (corresponding to *p* < 0.008) and randomly permuted the sign of each TRF N = 1000 times. We identified significant clusters as those with a cluster mass (summed *t*-values over electrodes∗time) larger than the size observed in permutation data at *p* < 0.05.
