# Supplementary material for: Neural encoding of linguistic features during natural sentence reading
Source: iScience. 2025 May 30;28(7):112798. doi: 10.1016/j.isci.2025.112798 (PMC12205611; doi:10.1016/j.isci.2025.112798)
Supplement: Document S1. Figures S1 and S2 [file mmc1.pdf]

iScience, Volume *28*

Supplemental information

Neural encoding of linguistic features during natural sentence reading

Vinay S. Raghavan and Lucas C. Parra

Supplementary Material

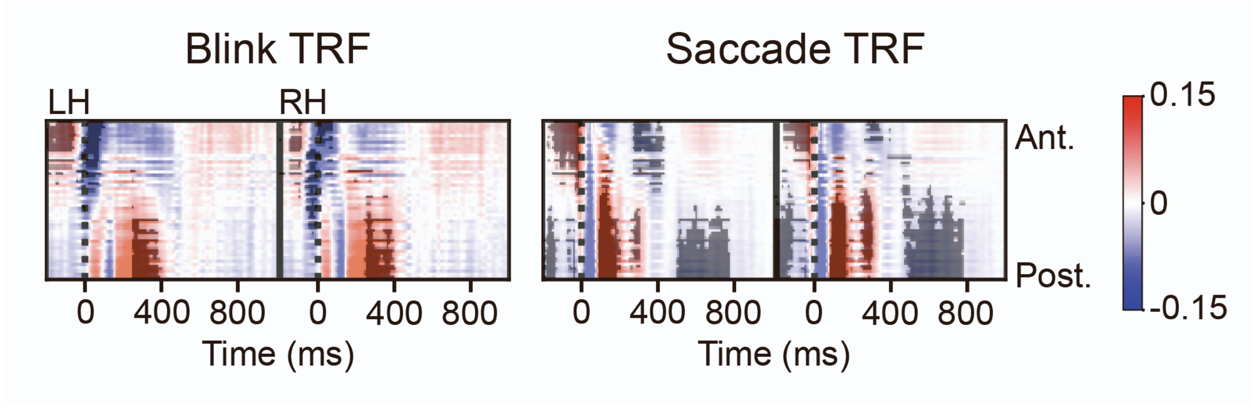

Supp Fig 1. Temporal response functions of blinks (left) and saccades (right). Left and right panels correspond to electrodes covering the left and right hemispheres, and vertical axes are different electrodes sorted from anterior to posterior scalp locations (top to bottom). Darker shading indicates significant clusters of electrodes and time points.

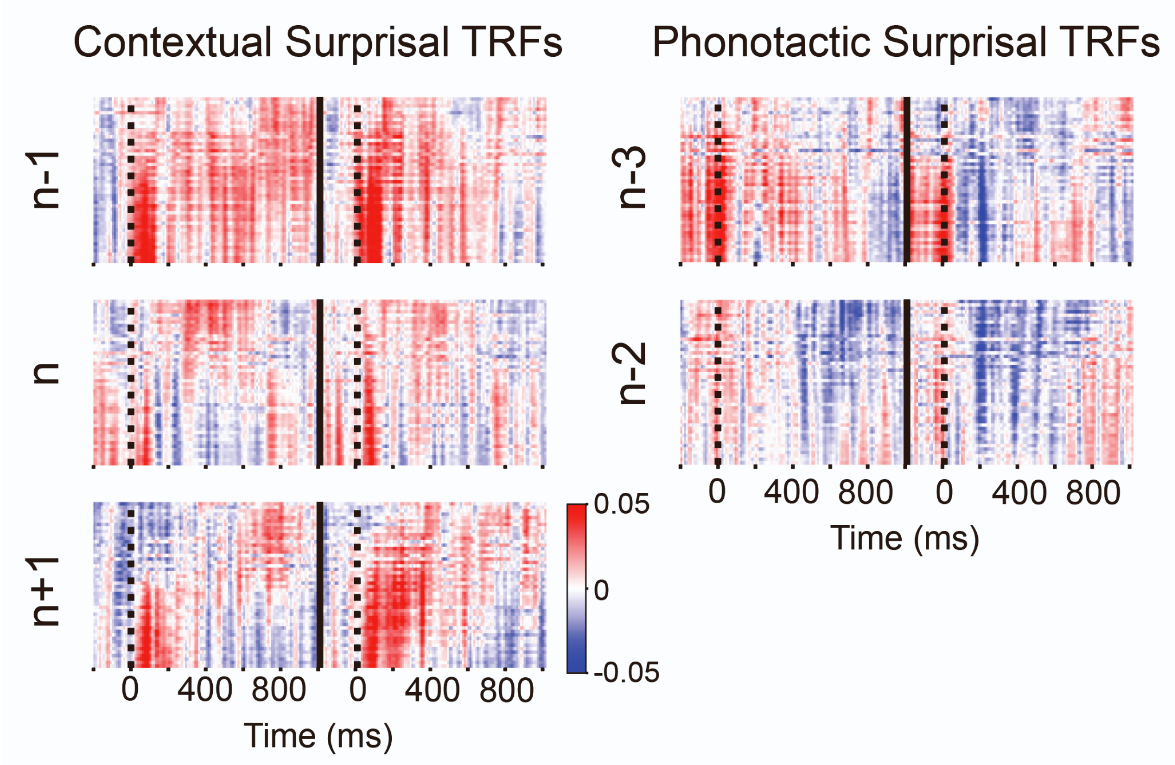

Supp Fig 2. Temporal response functions of linguistic encoding surrounding fixation. These TRFs were averaged to create those displayed in Fig 5. Left and right panels correspond to electrodes covering the left and right hemispheres, and vertical axes are different electrodes sorted from anterior to posterior scalp locations (top to bottom).
